# Expression of concern: MicroRNA-140-5p suppresses retinoblastoma cell growth via inhibiting c-Met/AKT/mTOR pathway

**DOI:** 10.1042/BSR-20180776_EOC

**Published:** 2020-08-21

**Keywords:** c-Met/AKT/mTOR signaling pathway, MiR-140-5p, retinoblastoma, tumorigenesis

The Editorial Office has been made aware of potential issues surrounding the scientific validity of this paper, hence has issued an expression of concern to notify readers whilst the Editorial Office investigates.

